# α-Syntrophin Modulates Myogenin Expression in Differentiating Myoblasts

**DOI:** 10.1371/journal.pone.0015355

**Published:** 2010-12-17

**Authors:** Min Jeong Kim, Sung Ho Hwang, Jeong A. Lim, Stanley C. Froehner, Marvin E. Adams, Hye Sun Kim

**Affiliations:** 1 Department of Biological Science, Ajou University, Suwon, Korea; 2 Department of Physiology & Biophysics, University of Washington, Seattle, Washington, United States of America; McMaster University, Canada

## Abstract

**Background:**

α-Syntrophin is a scaffolding protein linking signaling proteins to the sarcolemmal dystrophin complex in mature muscle. However, α-syntrophin is also expressed in differentiating myoblasts during the early stages of muscle differentiation. In this study, we examined the relationship between the expression of α-syntrophin and myogenin, a key muscle regulatory factor.

**Methods and Findings:**

The absence of α-syntrophin leads to reduced and delayed myogenin expression. This conclusion is based on experiments using muscle cells isolated from α-syntrophin null mice, muscle regeneration studies in α-syntrophin null mice, experiments in Sol8 cells (a cell line that expresses only low levels of α-syntrophin) and siRNA studies in differentiating C2 cells. In primary cultured myocytes isolated from α-syntrophin null mice, the level of myogenin was less than 50% that from wild type myocytes (*p*<0.005) 40 h after differentiation induction. In regenerating muscle, the expression of myogenin in the α-syntrophin null muscle was reduced to approximately 25% that of wild type muscle (*p*<0.005). Conversely, myogenin expression is enhanced in primary cultures of myoblasts isolated from a transgenic mouse over-expressing α-syntrophin and in Sol8 cells transfected with a vector to over-express α-syntrophin. Moreover, we find that myogenin mRNA is reduced in the absence of α-syntrophin and increased by α-syntrophin over-expression. Immunofluorescence microscopy shows that α-syntrophin is localized to the nuclei of differentiating myoblasts. Finally, immunoprecipitation experiments demonstrate that α-syntrophin associates with Mixed-Lineage Leukemia 5, a regulator of myogenin expression.

**Conclusions:**

We conclude that α-syntrophin plays an important role in regulating myogenesis by modulating myogenin expression.

## Introduction

The basic helix-loop-helix family of transcription regulatory factors is critical for proper development of skeletal muscle. MyoD and Myf5 are important for myogenic determination while myogenin and Mrf4 promote terminal differentiation of muscle precursor cells to mature skeletal muscle. Myogenin regulates the expression of a myriad of muscle specific genes and is necessary for normal muscle development [1]. Myogenin knockout mice have a severe muscle deformity at birth [2]–[3]. These mice accumulate myoblasts that are arrested in their terminal differentiation program. During normal muscle development, myogenin is not expressed in undifferentiating myoblasts but accumulates at the onset of differentiation [4]. Expression of myogenin is induced by factors that stimulate the myogenic program, such as the decrease in growth factors or presence of insulin-like growth factor-1 (IGF-1), a potent activator of differentiation [5]–[8].

α-Syntrophin has been widely studied for its role as a molecular adapter protein that recruits signaling proteins to the sarcolemmal dystrophin scaffold in mature muscle. α-Syntrophin binds ion channels [9]–[11], G-protein coupled receptors [12], water channels [13], kinases and associated proteins [14]–[17], and neuronal nitric oxide synthase [18].

Nearly all studies of skeletal muscle α-syntrophin have been performed using mature muscle. Very little is known about the role of α-syntrophin during early muscle development, even though myoblasts express α-syntrophin before terminal differentiation [19]. One study has shown that α-syntrophin localizes diacylglycerol kinase-ζ (DGKζ during several stages of myoblast fusion [19]. The most compelling evidence for a role of α-syntrophin in muscle development comes from investigation of muscle regeneration in a knockout mouse lacking of the α-syntrophin [20]. Regenerating skeletal muscle in this mouse showed several defects including fiber splitting, fiber type shifts, increased IGF-1 mRNA, and decreased muscle strength 7 days after regeneration.

The regeneration defects in the absence of α-syntrophin and the observation that α-syntrophin is present in undifferentiated myoblasts prompted us to investigate the role of α-syntrophin in muscle development. In this study we focus on the relationship between α-syntrophin and myogenin expression. We explore myogenin expression using *in vivo* and *in vitro* systems that vary in their level of α-syntrophin expression. Furthermore we investigate the temporal expression and spatial localization of α-syntrophin during myoblast differentiation to facilitate the understanding of α-syntrophin's role in muscle development.

## Materials and Methods

### Materials

Isoform-specific antibodies to syntrophins were previously prepared [21]. All other antibodies were purchased as follows; anti-myogenin from BD Bioscience (San Jose, CA) or Santa Cruz Biotechnology (Santa Cruz, CA), antibodies to dystrophin and β-actin from Sigma (St. Louis, MO), anti-MLL-5 antibody from Orbigen (San Diego, CA), antibodies to GAPDH and actin from Cell Signaling (Danvers, PA). α-Syntrophin-specific siRNA and control siRNA were from Santa Cruz Biotechnology (Santa Cruz, CA). Cardiotoxin (*Naja nigricollis*) was obtained from Calbiochem (La Jolla, CA). Rabbit TrueBlot beads and antibody were obtained from eBioscience (San Diego, CA). All the cell culture reagents, 0.25% Trypsin-EDTA, Lipofectamine 2000 and pcDNA 3.1 were from Invitrogen (Carlsbad, CA). RNA-SPIN™ total RNA extraction kit, Maxim™ RT premix kit, and other reagents for RT-PCR were from Intron Biotechnology (Seoul, Korea). Bradford assay kit for determine of protein concentration was from Bio-Rad (Hercules, CA).

### Cell culture

Primary skeletal muscle cells were isolated from the forelimbs and hindlimbs of neonatal mice (0–1 d old) from C57BL6 (wild-type), α-syntrophin knockout (α-KO) [22], α/β2-syntrophin knockout (aabb) [23] and α-syntrophin transgenic mice (FLA) [24] strains. The preparation was carried out according to previously described procedure [25] with slight modifications. Myoblasts were isolated from minced muscle tissue following dissociation with 0.25% trypsin-EDTA (final conc. 0.05%) in PBS for 45 min at 37°C. For myoblast enrichment, cells were passed through a strainer (80 µm pore size; BD Falcon, San Jose, CA) and pre-plated twice for 1 h each plating. The percentage of myoblasts could be maintained at greater that 70% by preplating the cells [26]. Cells then were seeded at a density of 3.0×10^5^ cells/ml in Dulbecco's modified Eagle's medium (DMEM) containing 10% fetal bovine serum (FBS) and 5% new-born calf serum for 1 day under 5% CO_2_ at 37°C. Then, cells were transferred into 2% horse serum (HS) containing DMEM for differentiation induction. C2 and Sol8 myogenic cells were seeded at a concentration of 1.0×10^4^ cells/ml. Cells were grown in 10% FBS containing DMEM (growth medium) for 3 days and induced to differentiate by transfer into 2% HS containing DMEM (Figure S1). Experimental procedures performed on mice were approved by the Institutional Animal Care and Use Committee of the University of Washington.

### Western blot analysis

Cells were rinsed twice with cold phosphate-buffered saline (PBS), harvested with a scraper and disrupted by ultrasonication in RIPA buffer, pH 7.4, containing 9.1 mM Na_2_HPO_4_, 1.7 mM NaH_2_PO_4_, 150 mM NaCl, 0.5% sodium deoxycholate, 0.1% SDS, 1 mM PMSF and 0.1% protease inhibitor cocktail (Sigma). Cell lysates were centrifuged at 15,000× *g* for 3 min to remove cell debris and the protein concentration of the supernatant determined using the Bradford assay. Equal amounts of total protein (20 g) were resolved by 4–15% gradient or 10% SDS-polyacrylamide gel electrophoresis and transferred onto polyvinylidene difluoride (PVDF) membrane (Millipore, MA). The membranes were incubated for 1 h in a blocking buffer (Tris-buffered saline containing 0.1% Tween-20 and 5% non-fat milk) and then incubated with primary antibodies (1∶1000) overnight at 4°C. After incubation with peroxidase conjugated secondary antibodies, the immune reactive protein bands were visualized by enhanced chemiluminescence (ECL) detection with Digital Luminescent Image Analyzer LAS-1000 (Fuji film, Japan). Band intensity was determined using Scion image software (Fredrick, MD).

### Immunofluorescence analysis

For immunolabeling, cells cultured on coverslips were fixed with 4% paraformaldehyde in PBS for 10 min and permeabilized with 0.5% Triton X-100 in PBS. After blocking with 10% normal goat serum for 10 min, cells were incubated with anti-α-syntrophin antibody (1∶50 dilution) for 1 h. Cells were then incubated with propidium iodide (PI) for staining nuclei, and a fluorescent secondary antibody (1∶100 dilution) for 40 min. The specimens were observed using a Zeiss LSM510 confocal laser scanning microscope.

### Separation of nuclear and cytoplasmic fractions

Cells were washed twice with cold PBS and harvested with a scraper, and then mixed with nuclear extraction buffer (20 mM HEPES, pH 7.5, containing 150 mM NaCl, 10% Glycerol, 1 mM EDTA, 0.5% Triton X-100, 1 mM Na_3_VO_4_, 10 mM NaF, 1 mM PMSF, and 0.01% of protease inhibitor cocktail) and incubated for 30 min on ice. The cell suspension was passed through a 23-gauge needle 30 times and then centrifuged at 1,000× *g* for 10 min. The pellet and supernatant from the centrifugation are referred to as the nuclear and cytoplasmic fractions, respectively. The nuclear fraction was washed 3 times with nuclear extraction buffer and then subjected to ultrasonication.

### Transfection of α-syntrophin specific small interference RNA (siRNA) or cDNA

For siRNA transfection, C2 cells (1.0×10^5^ cells/ml) were grown in 24-well tissue culture plates in an antibiotic-free DMEM containing 10% FBS for 24 h. Cells were transferred into Opti-MEM and then were transfected by incubation with control siRNA or α-syntrophin specific siRNA for 6 h. The transfected cells were transferred into differentiation medium (0 h) and cultured for the indicated times. For the over-expression of α-syntrophin, the full-length α-syntrophin cDNA [27] was cloned into the HindIII and BamHI site of pcDNA3.1. Sol8 cells (1.0×10^5^ cells/ml) were cultured in 24-well tissue culture plates in an antibiotic-free DMEM containing 10% FBS for 24 h. Cells of 90% confluence were incubated with α-syntrophin cDNA in pcDNA3 and used in transfection experiments with Lipofectamine 2000 according to the manufacturer's instructions.

### RNA preparation and reverse transcriptase (RT)-PCR

Total RNA was isolated from C2 or Sol8 cells using RNA-SPIN™ total RNA extraction kit. First strand cDNA synthesis was performed using 1 g RNA template with the Maxim™ RT premix kit according to the manufacturer's instructions. The synthesized cDNA was used as a template for amplification by PCR (30 cycles of 94°C for 30 sec, 59°C for 30 sec and 72°C for 30 sec for α-syntrophin and GAPDH; 30 cycles of 94°C for 30 sec, 55°C for 30 sec and 72°C for 30 sec for myogenin). The primer sequences used were: mouse α-syntrophin GCTAAAGAGGCGAAGATGG and TTATGATAGCTTCAAGGCCA; mouse myogenin, GCAGTGCCATCCAGTACATTGAGC and GGAAGGTGACAGACATATCCTCCAC; mouse GAPDH, ACCACAGTCCATGCCATCAC and TCCACCACCCTGTTGCTGTA.

### Cardiotoxin injection

Eight week old female C57BL6 (wild-type) and α-KO mice were anesthetized and 30 µl of cardiotoxin (10 µM in PBS) was injected into the left tibialis anterior (TA) muscle and the same volume of PBS was injected into the right TA as a control. At the indicated time points following injection, the TA muscles were isolated, frozen in liquid nitrogen and stored at −70°C until use. Each frozen muscle was added to 2.5 ml of a RIPA buffer and homogenized using a PT200E Polytron (Kinematica, Luzern, Switzerland). Muscle lysates were incubated on ice for 30 min, and then centrifuged 15,000 *g* for 3 min. The protein concentration of the supernatant was determined by Bradford assay (Bio-Rad) and the samples subjected to immunoblot analysis.

### Immunoprecipitation

Cells were harvested as above, lysed with a RIPA buffer and disrupted with an ultrasonicator. To remove cell debris, lysates were spun down at 15,000× g for 3 min. For pre-clearance, cell extracts (500 µg/500 µl) were incubated with 50 µl of TrueBlot beads for 30 min on ice. Anti-α-syntrophin antibody was then added to pre-cleared cell lysates and incubated overnight at 4°C. TrueBlot beads (50 µl) were added again to the extracts and incubated for 1 h at 4°C. The immune-complexes were collected by centrifugation, washed 3 times with PBS and separated with SDS-PAGE.

### Statistical analysis

For the statistical analysis of the myogenin level, two-tailed Student's unpaired *t* tests were performed. A value of *p*<0.01 was considered to be significant.

## Results

### Myogenin expression in mouse myocytes

**Figure 1:**
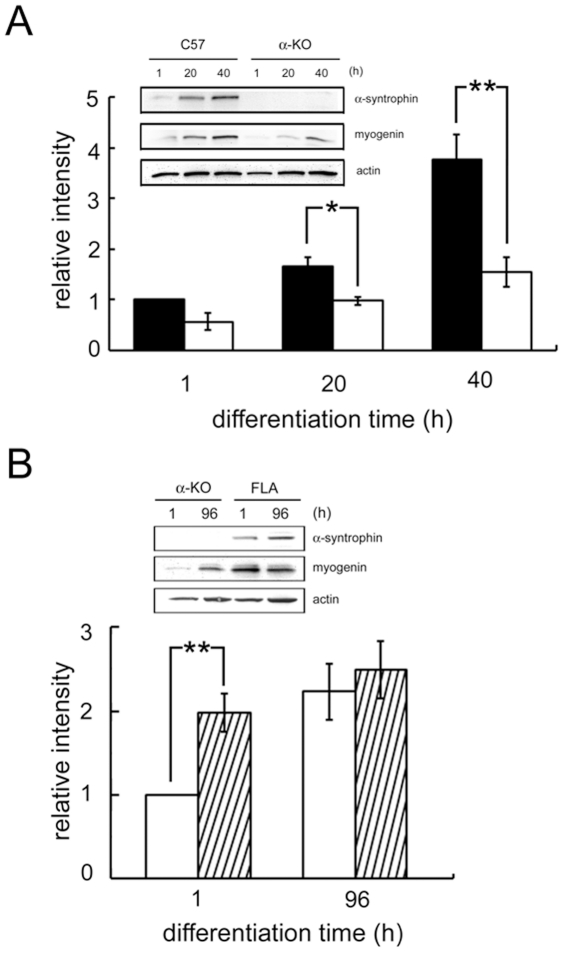
Expression of myogenin during myocyte differentiation. (A) Primary cultured skeletal muscle cells from wild-type (black bar) or α-KO (white bar) were harvested at the time points indicated after differentiation induction. The level of myogenin expression was determined by western blotting and the band intensity was quantified using Scion Image. The average band intensity of wild type myocytes at 1 h was defined as 1.00 to express the relative intensity of the remaining samples. Values are the mean ± S.E.M. of four independent experiments. (*p<0.01, **p<0.005). The inset shows a representative blot. (B) Similar experiments comparing the myogenin expression in α-KO (white bar) or the α-syntrophin transgenic, FLA (crosshatched bar). Values are expressed as mean ± S.E.M. of three independent experiments. (**p<0.005). Actin immunolabeling was used as a loading control.

In this work we investigated the role of α-syntrophin in muscle development focusing on its modulation of myogenin expression. For the first set of experiments, mononucleated skeletal myoblasts from α-syntrophin knockout (α-KO) or C57 wild-type mice were isolated from neonatal pups and grown in culture. In mitogen-rich growth medium, myoblasts proliferate with no prominent difference in morphology between the α-KO and controls. Upon transfer into low-mitogen differentiation medium, myoblasts fuse to produce multinucleated myotubes. Myogenin is first expressed during this differentiation into myotubes. We collected samples of the cultured muscle cells 1, 20, and 40 hrs after the addition of differentiation medium. Immunoblot analysis shows that in the undifferentiated myocytes (1 h), myogenin levels are relatively low, but as differentiation proceeds, the myogenin levels increase (Figure 1A). At 40 h after differentiation induction, myogenin levels are approximately four times the level at 1 h. Myogenin expression in α-KO cells also increases during differentiation but is consistently lower than that of C57 cells. At 40 h after differentiation induction, myogenin levels in the α-KO samples are less than half those of the C57 samples.

To strengthen the correlation between syntrophin and myogenin expression, we compared myogenin levels of α-KO myocytes with those of myocytes derived from α-syntrophin-transgenic mice (FLA). These mice over-express α-syntrophin in striated muscle and have been bred onto the α-syntrophin KO background so that the only α-syntrophin expressed is derived from the transgene [24]. The level of myogenin in the FLA myoblasts is more than twice that of α-KO at the first (1 h) time point of differentiation (Figure 1B). After 96 h of differentiation, when myoblasts fusion peaks, there is no significant difference in myogenin expression. These results indicate that α-syntrophin expression is correlated with the expression of myogenin during the early differentiation of cultured skeletal muscle cells.

### Myogenin expression in regenerating mouse muscle

**Figure 2:**
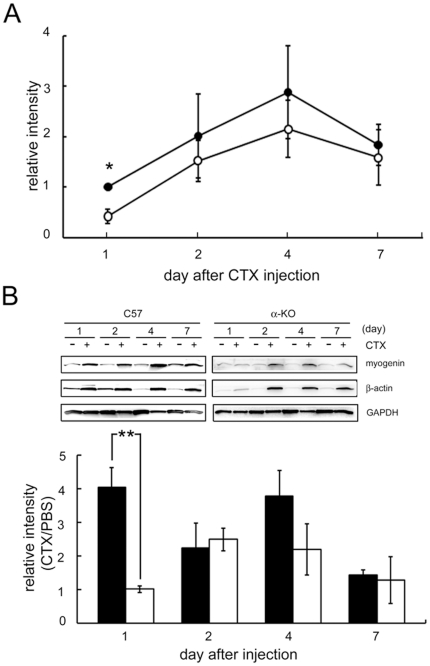
Expression of myogenin in regenerating muscle. (A) The tibialis anterior (TA) muscles of wild type (-•-) and α-KO (-○-) mice were injected with cardiotoxin (CTX) to induce regeneration. After the indicated number of days following injection, muscles were harvested and myogenin expression was assayed by western blot and the band intensity was quantified using Scion Image. The intensity of the myogenin band from the wild type at 1 day is represented as 1.00 and the other bands are expressed relative to this band. (B) Ratio of myogenin expression in the CTX injected to the contralateral PBS-injected TA muscle at the indicated days after injection. Values are expressed as the mean ± S.E.M. of three independent experiments. (*p<0.005, **p<0.01). The ratio of myogenin band intensity from α-KO at 1 day was represented as 1.00. β-Actin was used as a regeneration marker and GAPDH was used as a loading control. The black and white bars indicate wild type and α-KO, respectively.

Based on our findings in cultured primary myoblasts, we investigated the relationship between α-syntrophin and myogenin expression using an *in vivo* system. Myogenin is down-regulated in mature muscle fibers [28]. Therefore, we investigated myogenin expression in regenerating muscle. We induced regeneration by injecting cardiotoxin directly into the muscle of 8-week-old control and α-KO mice. Cardiotoxin was injected into the tibialis anterior (TA) muscle and the contralateral TA was injected with PBS as a control. Since previous studies showed that myogenic differentiation begins within 3 days after cardiotoxin injection [29], we measured myogenin expression during the first week following injection. Although the pattern of myogenin expression is similar for control and α-KO mice (present from day 1, peaking at day 4, then diminishing at day 7) the myogenin levels in α-KO muscle are lower than the control at each time point (Figure 2A). Myogenin is also expressed at much lower levels in the PBS-injected TA muscle of both C57 and α-KO (Figure 2B). When the myogenin levels in the regenerating muscle are normalized to the contralateral PBS control, the difference in early expression becomes clear (Figure 2B). One day after injection, the ratio of myogenin in cardiotoxin-treated to PBS injected muscle in α-KO mice is less than 25% that of control mice. This implies that regeneration is delayed in α-KO mice compared with wild-type mice. Supporting this conclusion is the expression of β-actin, a regeneration marker in skeletal muscle [30]. β-Actin is expressed at much lower levels in α-KO mice compared with wild-type mice at 1 day after cardiotoxin injection (Figure 2B).

### α-Syntrophin modulates myogenin expression in myogenic cell lines

**Figure 3:**
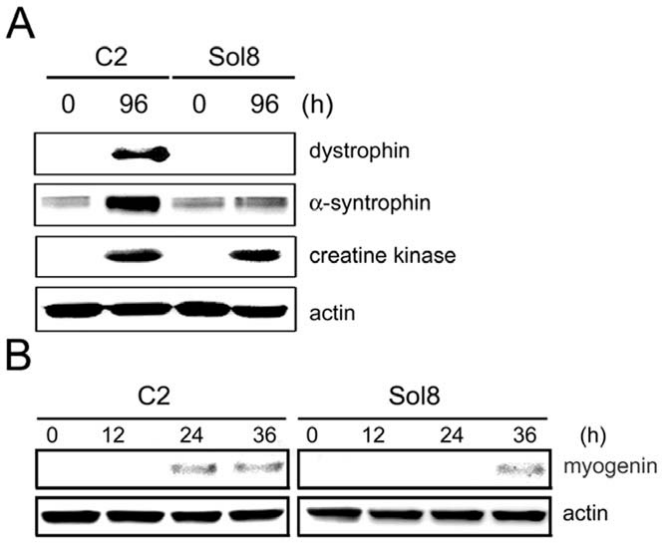
Myogenin expression in Sol8 and C2 cell lines. (A) The expression of dystrophin, α-syntrophin, and creatine kinase was assessed by western blotting in C2 and Sol8 myocytes before (0 h) and after (96 h) differentiation to myotubes. (B) Myogenin expression assessed by western blotting at the indicated time points following differentiation induction. Actin was used as a loading control. Results are representative of three independent experiments.

To complement these studies of α-syntrophin and myogenin expression *in vivo*, we used cultured cell lines that could be further manipulated. Both the C2 cell line, isolated from adult mouse skeletal muscle [31], and the Sol8 line isolated from mouse oxidative (soleus) muscle [32] were used for these studies. One difference between these lines is that Sol8 cells do not express dystrophin [33]. In Duchenne muscular dystrophy the absence of dystrophin leads to a large reduction of the dystrophin associated proteins [34]–[36]. Sol8 cells do not express α-sarcoglycan, one of the dystrophin-associated proteins [33], but there have been no reports of the expression of syntrophins in these cells. Therefore, we examined the expression of α-syntrophin during the differentiation of C2 and Sol8. Before differentiation induction (0 h), the level of α-syntrophin is similar in both cell lines. However, the level of α-syntrophin dramatically increases in the differentiating C2 myotubes (Figure 3A). In contrast, the level of α-syntrophin in Sol8 cells does not increase during differentiation. As a control, the level of creatine kinase, a muscle specific protein and late differentiation marker, is similar in both lines after 96 h (Figure 3A). We also examined the expression of myogenin during differentiation. At time 0 h myogenin is undectable in both C2 and Sol8 myoblasts. However, 24 h after differentiation induction myogenin can be detected in C2 cells but not in Sol8 cells. Myogenin was eventually detected at 36 h in Sol8 cells indicating a delay in expression of approximately 12 h compared to C2 cells (Figure 3B).

**Figure 4:**
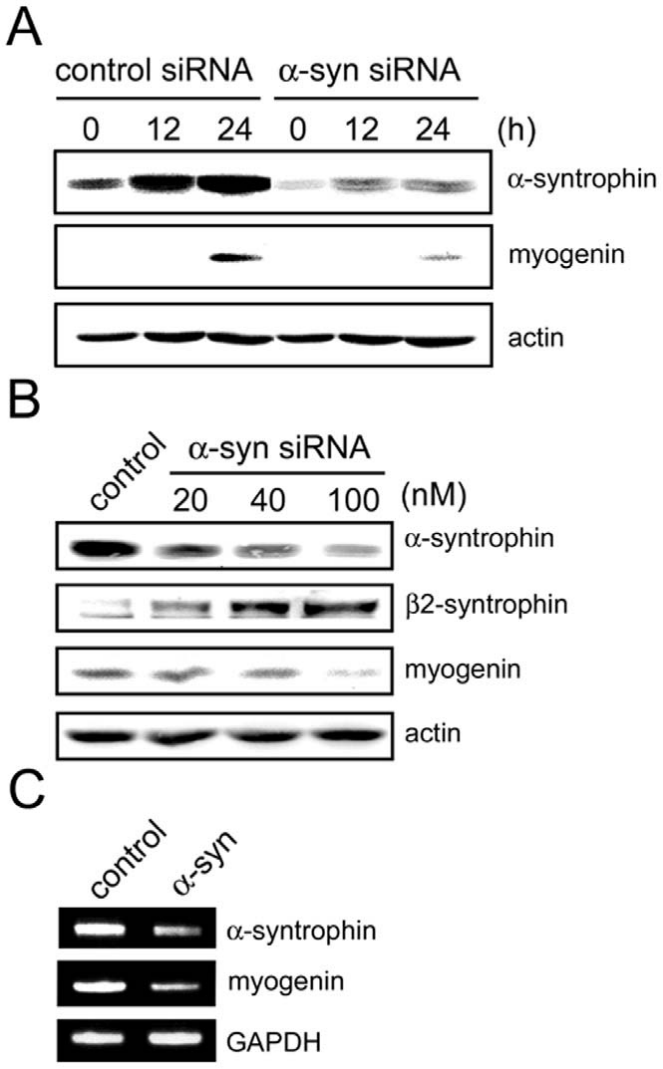
siRNA knock-down of α-syntrophin suppresses myogenin expression in C2 cells. (A) C2 myoblasts were transfected with control siRNA or α-syntrophin-specific siRNA. Cells were transferred into differentiation medium and harvested at the indicated times. The expression of α-syntrophin or myogenin was assessed by western blotting. (B) Cells were transfected with increasing doses of control or α-syntrophin-specific siRNA and harvested 24 h after differentiation induction. Cell lysates were analyzed for expression of α-syntrophin and myogenin by western blotting. Actin was used as a loading control. (C) C2 cells transfected with control or α-syntrophin-specific siRNA (100 nM) were assayed for mRNA levels of α-syntrophin and myogenin by RT-PCR. GAPDH was used as a control. Results are representative of at least three independent experiments.

We also performed a knock-down experiment by transient transfection with an α-syntrophin-specific siRNA. C2 myoblasts were transfected with control siRNA or α-syntrophin-specific siRNA for 6 h. The transfected cells were transferred into differentiation medium and harvested for analysis at the indicated times (Figure 4A). The expression of α-syntrophin is greatly reduced in the α-syntrophin-specific siRNA-transfected cells (Figure 4), compared with the control siRNA-transfected cells. The control cells show an increase in α-syntrophin expression during differentiation similar to that of non-transfected cells (see Figure 3). Myogenin is detected at 24 h in both control and α-syntrophin specific siRNA-transfected cells, but the level of myogenin in the α-syntrophin siRNA-transfected cells is significantly reduced (approximately 20% of control). The reduction of myogenin by α-syntrophin siRNA is dose-dependent. The expression of myogenin decreases with increasing dose of the transfected siRNA (Figure 4B).

The lower expression of myogenin could be caused by reduced levels of transcription or by post-transcriptional mechanisms. To differentiate between these possibilities, we measured the relative myogenin mRNA levels using RT-PCR (Figure 4C). The myogenin mRNA level is reduced in the α-syntrophin siRNA-transfected C2 cells. This result indicates that the reduction of myogenin in the α-syntrophin siRNA-transfected cells most likely occurs at the transcriptional level.

**Figure 5:**
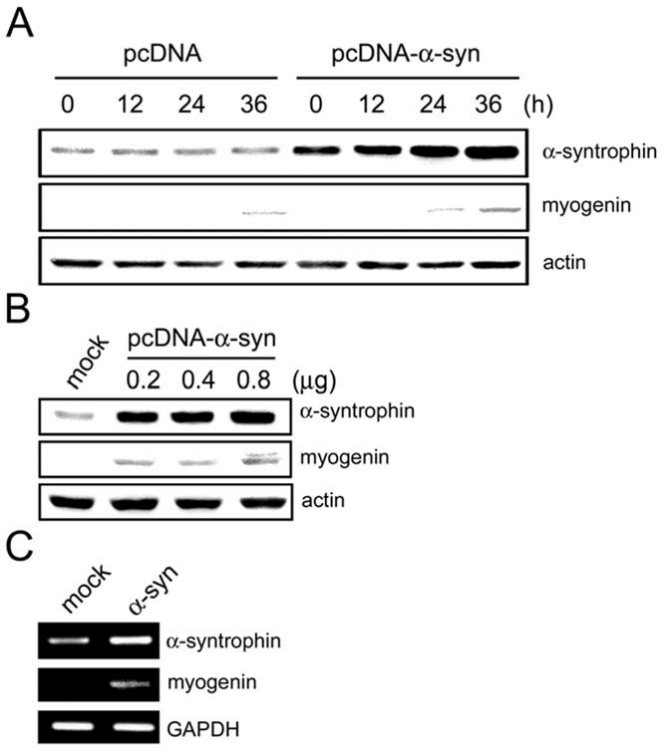
Over-expression of α-syntrophin promotes myogenin expression in Sol8 cells. (A) Sol8 myoblasts were transfected with pcDNA (control) or pcDNA containing full-length α-syntrophin DNA. Transfected cells were transferred to differentiation medium, harvested at the indicated time points, and the expression of α-syntrophin and myogenin determined by western blotting. (B) Sol8 cells were transfected with increasing doses of pcDNA (mock) or pcDNA containing full-length α-syntrophin DNA and incubated in differentiation medium for 24 h. Expression of α-syntrophin and myogenin was assessed by western blotting. Actin was used as a loading control. (C) Transfected Sol8 cells (using 0.8 µg of DNA) were cultured for 24 h in a differentiation medium. The mRNA levels of α-syntrophin and myogenin were assayed by RT-PCR. GAPDH was used as a control. Results are representative of at least three independent experiments.

Based on the strong relationship between α-syntrophin and myogenin expression, we hypothesized that artificially increasing the expression of α-syntrophin in Sol8 cells could increase myogenin expression to that observed in C2 cells (see Figure 3A). We tested this hypothesis by transfecting Sol8 myoblasts with pcDNA or pcDNA-α-syntrophin. After transferring the transfected cells into differentiation medium (0 h), cells were harvested at the indicated time points and the expression of α-syntrophin and myogenin assayed by immunoblotting (Figure 5). The expression level of α-syntrophin increases with differentiation time in pcDNA-α-syntrophin-transfected cells but remains constant in the control (pcDNA-transfected) cells (Figure 5A). Myogenin is detected at the 24 h point in pcDNA-α-syntrophin-transfected cells; 12 h earlier than in pcDNA-transfected cells or in non-transfected control Sol8 cells (see Figure 3B). Myogenin expression increases in a dose-dependent manner with increasing concentration of pcDNA-α-syntrophin (Figure 5B). RT-PCR analysis shows that transfection of pcDNA-α-syntrophin increases the myogenin mRNA levels. Together these data confirm our hypothesis that increasing levels of α-syntrophin in Sol8 cells leads to a corresponding increase in myogenin levels and that this increase is most likely due to an increase in transcription.

**Figure 6:**
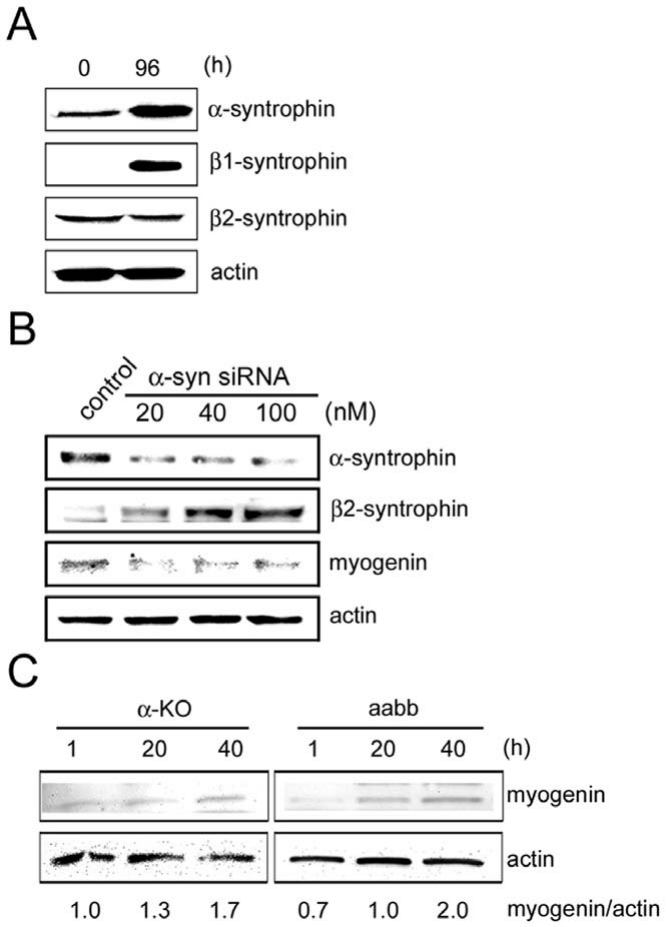
β2-Syntrophin does not contribute to the regulation of myogenin expression. (A) The expression of β1-syntrophin, β2-syntrophin, as well as α-syntrophin was determined by western blotting in C2 undifferentiated myoblasts (0 h) and differentiated myotubes (96 h). Actin represents a loading control. (B) C2 myoblasts were transfected with increasing dose of control siRNA or α-syntrophin-specific siRNA and harvested 24 h after differentiation induction. Expression of α-, β1- and β2-syntrophins was determined with previously characterized antibodies. Actin represents a loading control. (C) Primary cultured skeletal muscle cells from α-KO and aabb (α/β2-syntrophin double knockout) were harvested at the indicated time after differentiation induction. The expression level of myogenin was determined western blotting and band intensity was measured using Scion Image software. The band intensities were expressed as the ratio of myogenin to actin. Results are representative of at least three independent experiments.

Although α-syntrophin is a dominant isoform in skeletal muscle, other types of syntrophin are also expressed in skeletal muscle. Therefore, we examined a possibility that these other isoforms could compensate for the absence of α-syntrophin in regulating myogenin expression in C2 cells. In the differentiating myoblasts, β1-syntrophin is detected only in the fully differentiated myotubes (96 h) but not in the undifferentiated myoblasts (0 h). In contrast, the expression of β2-syntrophin occurs both in myoblasts and myotubes without significant changes in the level (Figure 6A). We examined expression of β2-syntrophin in the α-syntrophin-specific siRNA-transfected cells (Figure 6B). β2-Syntrophin is increased with decreasing level of α-syntrophin by transfection. However, the increased β2-syntrophin does not prevent the decrease of myogenin. This result implies that β2-syntrophin does not compensate for the absence of α-syntrophin in regard to myogenin expression. To confirm the observation, we compared myogenin expression in the primary cultured myoblasts from the α-KO and α/β2-syntrophin double knockout mouse (aabb) [23]. There is no significant difference in the expression of myogenin differentiation of myoblasts derived from these mice (Figure 6C).

### Nuclear localization of α-syntrophin

**Figure 7:**
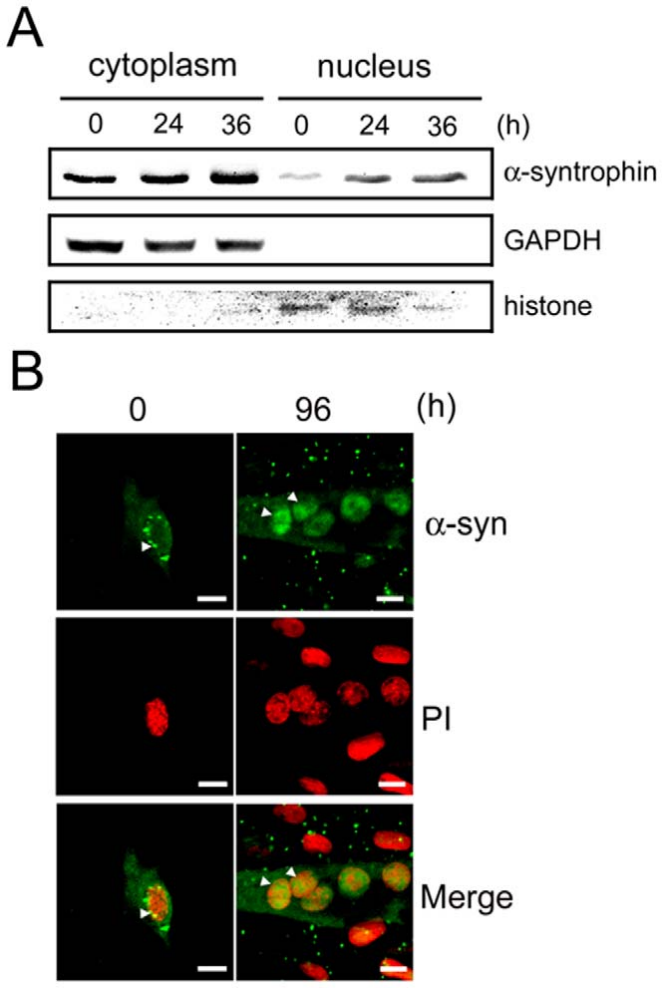
α-Syntrophin is localized in the nuclei of differentiating C2 myotubes. (A) The nuclear fraction was separated from the cytoplasmic fraction of C2 cells at the indicated times after differentiation induction. Western blotting shows that α-syntrophin is present in both fractions but expression increases in the C2 nuclear fraction with differentiation. GAPDH and histone were used as markers for cytoplasm and nuclear fractions, respectively. (B) Immunofluorescence of α-syntrophin in undifferentiated myoblasts (0 h) and differentiated myotubes (96 h) of C2 cells shows its presence in the nucleus. Propidium iodide (PI) staining marks nuclei. Arrowheads indicate nuclear localization of α-syntrophin in C2 myoblasts and myotubes. Scale bar  = 10 µm.

If α-syntrophin directly modulates myogenin transcription, one would expect to find α-syntrophin in the myocyte nucleus. In mature muscle, α-syntrophin is primarily localized at the sarcolemma in association with dystrophin [37]–[38]. However, α-syntrophin has been observed in the nuclear region of cultured muscle cells [39]. We used both biochemical and immunofluorescence techniques to examine the subcellular localization of α-syntrophin in C2 and Sol 8 cells. Cytoplasmic and nuclei enriched fractions were prepared by centrifugation from cell samples acquired at 12 hr increments following induction of differentiation. As expected, α-syntrophin is mainly present in the cytoplasmic fraction, but α-syntrophin is also present in the nuclear fraction of the C2 myocytes (Figure 7A). The amount of α-syntrophin in the nuclear fraction increased during differentiation of the C2 cells. In contrast, only very low levels of α-syntrophin are observed in the nuclear fraction of Sol8 cells (Figure S2). Immunofluorescence studies of both C2 and Sol8 cells show that while α-syntrophin is found throughout the cytoplasm, areas of concentration are present in the nucleus of C2 myoblasts. The nuclear α-syntrophin increases in the differentiated C2 myotubes (arrowheads in Figure 7B). In contrast, very little nuclear α-syntrophin is observed in the Sol8 myoblasts and myotubes (Figure S2).

### α-Syntrophin associates with MLL-5

**Figure 8:**
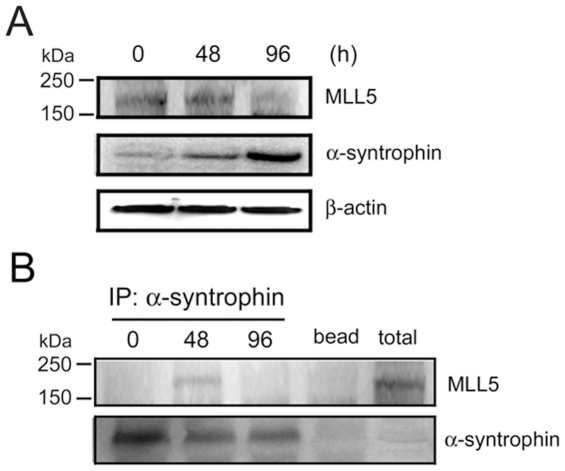
α-Syntrophin association with MLL5. (A) C2 myoblasts were induced to differentiate and the expression of MLL5 and α-syntrophin was assessed by western blot at the indicated time points. β-Actin is used as a loading control. (B) At the indicated differentiation times, C2 cells were harvested and the extracts immunoprecipitated using an anti-α-syntrophin antibody. MLL5 amount was assessed by western blot. The “bead” lane refers to a control lacking the IP antibody (α-syntrophin) and the “total” lane refers to a cell extract collected at 0 h. Results are representative of three independent experiments.

The expression of muscle regulators, including myogenin, is regulated by the histone methyltransferase, myeloid/lymphoid or mixed-lineage leukemia 5 (MLL5) [40]. Since our results showed that α-syntrophin expression also regulates myogenin expression and that α-syntrophin is present in the myocyte nucleus, we hypothesized that α-syntrophin may be associated with MLL5 in these cells. To test this hypothesis we compared the expression of MLL5 and α-syntrophin in differentiating C2 myocytes (Figure 7A). MLL5 is detected in undifferentiated myoblasts (0 h) and at 48 h, but is absent 96 h after differentiation induction (Figure 8A). Also at the indicated time points we immunoprecipitated α-syntrophin and tested the immunocomplexes for MLL5 (Figure 8B). The MLL5 band is detected only at the 48 h time point implying that α-syntrophin and MLL5 associate transiently during the time when myoblasts are undergoing differentiation. This result suggests that the interaction of MLL5 and α-syntrophin is regulated in a time-specific manner during myogenic differentiation.

## Discussion

In mature skeletal muscle, α-syntrophin associates with dystrophin to provide scaffolding that localizes to the sarcolemma various channels, kinases and signaling proteins [9]–[11], [13]–[17]. The α-syntrophin scaffolding has been extensively studied in mature skeletal muscle both at the neuromuscular junction and on the sarcolemma [41]. However, the function of α-syntrophin during early development of skeletal muscle has not been previously characterized. In this work we find that in addition to sarcolemmal scaffolding, α-syntrophin functions to modulate the expression of myogenin in differentiating muscle. We used a variety of experiments to demonstrate that both the time-course and the amount of myogenin expression depend on the level of α-syntrophin expression.

Our results show that the absence of α-syntrophin leads to reduced and delayed expression of myogenin. We observed this effect in differentiating myoblasts from primary cell cultures isolated from the α-syntrophin null mice. Similarly we observed reduced and delayed expression of myogenin in cultures of α-syntrophin siRNA treated C2 cells. The siRNA results are particularly interesting because they show that the level of myogenin expression is dependent on the concentration of siRNA used. During differentiation of Sol8 cells (a cell line that expresses only low levels of α-syntrophin compared to those in C2 cells), myogenin expression is reduced and delayed. Taken together, these experiments demonstrate that reduced α-syntrophin leads to reduced expression of myogenin. We have also observed reduced myogenin levels *in vivo* during regeneration of muscle from α-syntrophin null mice. This finding is consistent with a published report of muscle performance in the regenerating muscle of α-KO mice [20]. This study found that shortly after regeneration (7 days after cardiotoxin injection) the grip strength of α-syntrophin knockout mice was significantly less than that of control mice. This observation implies that regeneration is slower in the absence of α-syntrophin, a finding consistent with our observed reduced and delayed myogenin expression in differentiating myoblasts.

Conversely, we find that over-expression of α-syntrophin enhances myogenin expression. Primary myocytes isolated from transgenic mice expressing high levels of α-syntrophin (on the α-syntrophin null background) show a high level of myogenin expression during differentiation. Likewise, differentiating Sol8 myocytes have much higher levels of myogenin when transfected with pcDNA-α-syntrophin, a construct expressing α-syntrophin. This increase in myogenin expression is also dose-dependent. These *in vitro* and *in vivo* experiments clearly demonstrate a direct correlation between the expression of α-syntrophin and myogenin in differentiating myoblasts. This correlation is most pronounced during the early stages of muscle differentiation. Muscles of α-syntrophin null mice mature and function normally in the adult mouse [22], [42]. However, functional muscle regeneration is slower in the absence of α-syntrophin [20]. These observations suggest that α-syntrophin is not required for myogenin expression and myoblast differentiation. Rather, α-syntrophin modulates myogenin expression during a critical time in the early stages of differentiation.

To address how α-syntrophin modulates myogenin expression we determined myogenin mRNA levels during differentiation. In α-syntrophin siRNA-treated C2 myocytes, the levels of myogenin mRNA are reduced, similar to the protein levels. Furthermore, we observed that in Sol8 cells overexpressing α-syntrophin, myogenin mRNA levels were increased. These results suggest that α-syntrophin levels modulate the amount of myogenin mRNA. This modulation most likely occurs via an affect on myogenin transcription, although we have not ruled out a change in myogenin mRNA stability/turnover. However, we do find α-syntrophin in the nuclei of differentiating myocytes at a time consistent with transcriptional regulation of myogenin. Nuclear localization of α-syntrophin has been previously reported in C2C12 muscle cells [39]. Furthermore, the level of α-syntrophin in the nucleus of C2C12 myocytes increases following induction of muscle differentiation [39]. The primary sequence of α-syntrophin does not contain a conventional nuclear localization signal sequence and we do not know how α-syntrophin is directed to the nucleus. However, since α-syntrophin is present in the nucleus and is known to bind multiple proteins involved in cell signaling, we propose that α-syntrophin is involved in nuclear signaling that results in modulation of myogenin transcription.

One pathway that regulates myogenin transcription involves the histone methyltransferase MLL5. MLL5 is part of a multiprotein complex that regulates transcription at the epigenetic level by mechanisms such as chromatin remodeling and histone modification [43]–[44]. Expression of several regulators of muscle differentiation, including myogenin, are impaired when MLL5 expression is reduced by siRNA [40]. We hypothesized that syntrophin could be part of the multiprotein complex associated with MLL5. We find that MLL5 co-purifies with α-syntrophin in the specific stages of differentiation when differentiation of myoblasts is being initiated. Thus, α-syntrophin may regulate myogenin expression by association with MLL5, perhaps providing a nuclear scaffold to organize a multiprotein signaling complex.

Our results demonstrate a novel function for α-syntrophin as a positive regulator early in the differentiation of myoblasts. α-Syntrophin enhances myoblast differentiation and muscle development by promoting myogenin expression. We have shown that α-syntrophin is part of a nuclear protein complex that includes MLL5, a known regulator of myogenin expression. Future studies will focus on identifying other proteins in the complex and other pathways whereby α-syntrophin modulates muscle protein expression.

## Supporting Information

Figure S1 Myoblast fusion in C2 cells and Sol8 cells. Both C2 and Sol8 myoblasts were induced to differentiate by transferring into 5% horse serum-containing differentiation medium (time 0 h). (A) C2 and Sol8 cells were stained with hematoxylin and observed by microscopy at the indicated times following differentiation induction. Scale Bar  = 20 µm. (B) Degree of cell fusion in 10 randomly chosen fields was determined at the indicated culture times. Values are expressed as mean ± S.E.M. of three independent experiments. (**p<0.01). Cells were considered to be fused only if there was clear cytoplasmic continuity and at least three nuclei present within the myotubes.
(TIF)

Figure S2 The subcellular localization of α-syntrophin in Sol8. (A) The nuclear fraction was separated from the cytoplasmic fraction in Sol8 cells at the indicated times after differentiation induction. The level of α-syntrophin was assessed by western blot. GAPDH and histone were used as markers for cytoplasm and nuclear fractions, respectively. (B) Immunofluorescence of α-syntrophin in undifferentiated myoblasts (0 h) and differentiated myotubes (96 h) of Sol8 cells shows minimal presence of α-syntrophin in the nucleus. PI staining marks nuclei. Scale bar  = 10 µm.
(TIF)
