# Supplementary figures and images for: α-Syntrophin Modulates Myogenin Expression in Differentiating Myoblasts
Source: PLoS One. 2010 Dec 17;5(12):e15355. doi: 10.1371/journal.pone.0015355 (PMC3003685; doi:10.1371/journal.pone.0015355)

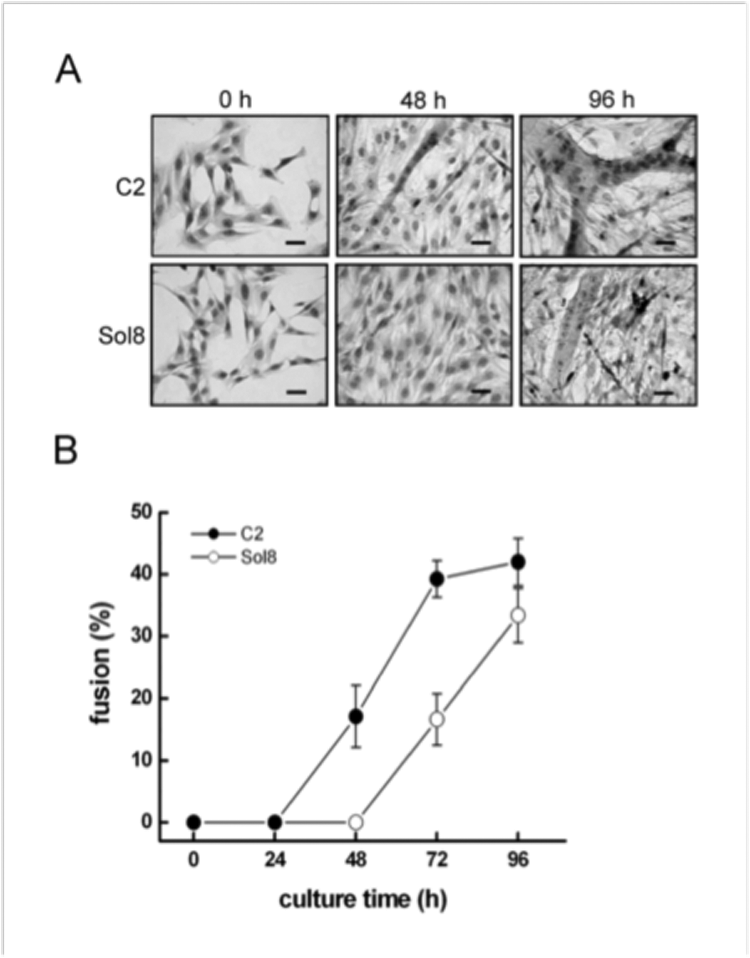

Supplement: Figure S1 — Myoblast fusion in C2 cells and Sol8 cells. Both C2 and Sol8 myoblasts were induced to differentiate by transferring into 5% horse serum-containing differentiation medium (time 0 h). (A) C2 and Sol8 cells were stained with hematoxylin and observed by microscopy at the indicated times following differentiation induction. Scale Bar = 20 µm. (B) Degree of cell fusion in 10 randomly chosen fields was determined at the indicated culture times. Values are expressed as mean ± S.E.M. of three independent experiments. (**p<0.01). Cells were considered to be fused only if there was clear cytoplasmic continuity and at least three nuclei present within the myotubes. (TIF) [file pone.0015355.s001.tif]

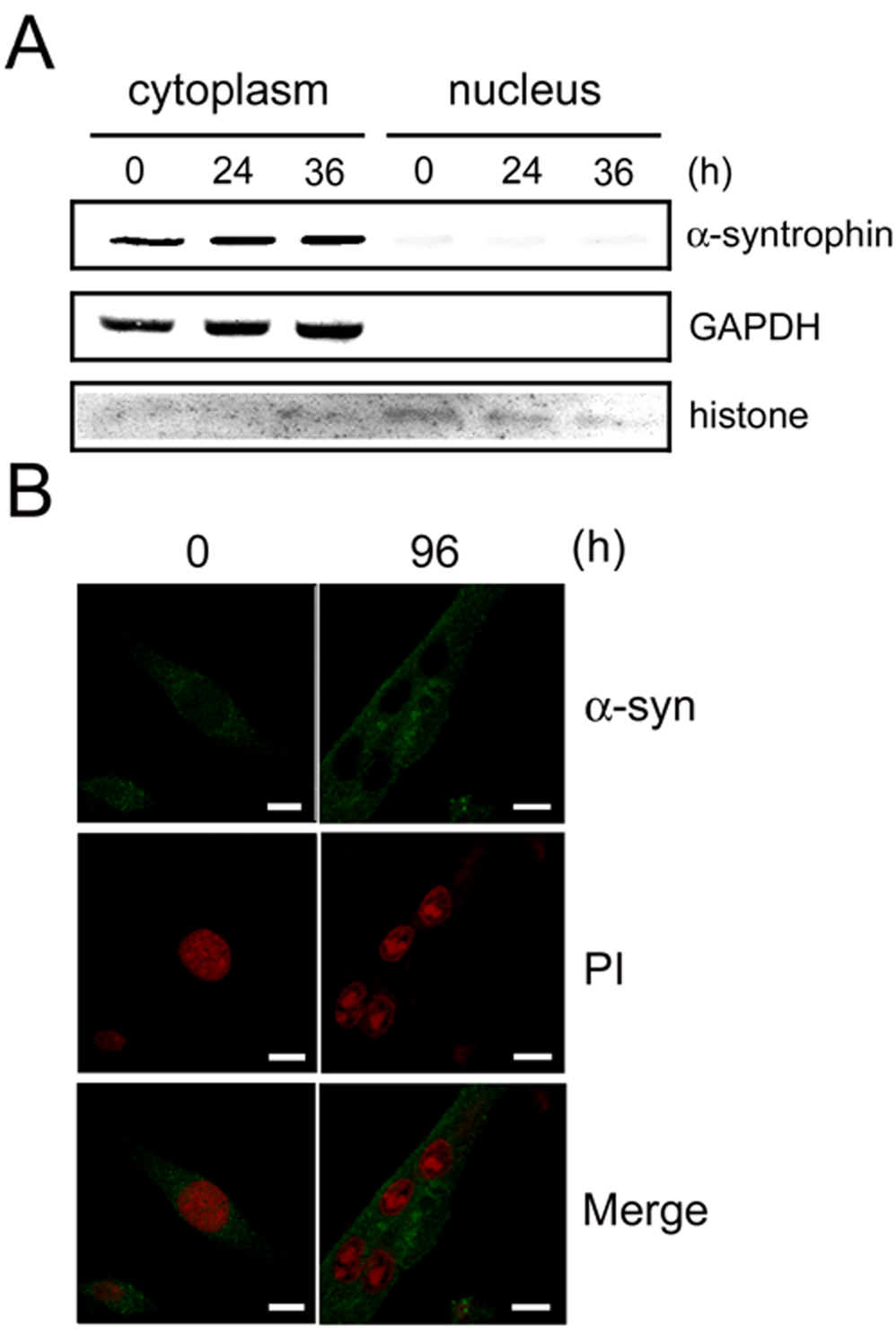

Supplement: Figure S2 — The subcellular localization of α-syntrophin in Sol8. (A) The nuclear fraction was separated from the cytoplasmic fraction in Sol8 cells at the indicated times after differentiation induction. The level of α-syntrophin was assessed by western blot. GAPDH and histone were used as markers for cytoplasm and nuclear fractions, respectively. (B) Immunofluorescence of α-syntrophin in undifferentiated myoblasts (0 h) and differentiated myotubes (96 h) of Sol8 cells shows minimal presence of α-syntrophin in the nucleus. PI staining marks nuclei. Scale bar = 10 µm. (TIF) [file pone.0015355.s002.tif]
